# Mayer-Rokitansky-Kuster-Hauser Syndrome: A rare case report from Nepal

**DOI:** 10.1016/j.amsu.2022.104725

**Published:** 2022-09-16

**Authors:** Umesh Ray, Subodh Adhikari, Roman Dhital, Sushant Shrestha, Sangam Shah, Sunil Poudel, Sanjit Kumar Sah, Roshan Gami, Abhishek Adhikari, Bishnu Gautam

**Affiliations:** a Institute of Medicine, Tribhuvan University, Maharajgunj, 44600, Nepal; b Department of Gynecology and Obstetrics, Lumbini Provincial Hospital, Lumbini, 32914, Nepal

**Keywords:** MRKHS, Nepal, Mayer-Rokitansky-Kuster-Hauser syndrome

## Abstract

**Introduction:**

Mayer-Rokitansky-Kuster-Hauser Syndrome (MRKHS) is a rare congenital disorder with an incidence of 1 in 5000 females. It is characterized by uterovaginal aplasia with normal secondary sexual characteristics and genetic karyotype 46XX. The exact etiology of MRKH syndrome is not known.

**Case presentation:**

We report a case of type 2 MRKHS with agenesis of left kidney.

**Discussion:**

The diagnosis of MRKH mainly depends on imaging study. Transabdominal ultrasonography is the first line investigation but abdomino-pelvic MRI gives more precise and clear information than the prior. So, we suggested our patient to do MRI even though she had done ultrasonography earlier. The differential diagnosis includes congenital vaginal agenesis, low transverse vaginal septum, androgen insensitivity, and imperforate hymen.

**Conclusion:**

This case presents that MRKH syndrome can occur with normal endocrine function and secondary sexual characteristics. Surgical correction by creating a neovagina is a good treatment method in young females for sexual intercourse.

## Highlights

• MRKH syndrome can occur with normal endocrine function and secondary sexual characteristics.
• Surgical correction by creating a neovagina is a good treatment method in young females for sexual intercourse.

## 1 Introduction

Mayer-Rokitansky-Kuster-Hauser Syndrome (MRKHS) or Mullerian dysgenesis is a rare congenital disorder with an incidence of 1 in 5000 females. MRKHS is characterized by uterovaginal aplasia with normal secondary sexual characteristics and genetic karyotype 46XX [1]. They are of two types: type 1 having only uterovaginal agenesis and type 2 having uterovaginal agenesis with anomalies in fallopian tube, kidney, spine, heart and other organ amenorrhea and painful sexual intercourse [1]. The exact etiology of MRKH syndrome is not known. Previously, drugs like diethylstilbestrol (DES) and thalidomide were said to have teratogenic causes for MRKH syndrome [2]. Counseling of the patient and neo vagina creation for sexual intercourse is the mainstay for the management. Here, we report a case of type 2 MRKHS with agenesis of left kidney. This case has been reported as per SCARE 2020 criteria [3].

## 2 Case report

A 25-year-old married female presented to our hospital with the chief complaints of primary amenorrhea and painful sexual intercourse. She had normal secondary sexual characteristics. Ultrasonography was done when she was 14 years old where she came to know that she had no uterus and vagina. Her mother and her sister had menarche at 12 and 13 years, respectively. There was no history of amenorrhea in the first and second-degree relatives. Her mother confirmed no known exposure to any medication or maternal illness during pregnancy. Other parts of the history were noncontributory. She is a non-smoker, non-alcoholic and consumes mixed diet. She had no past medical history and has not undergone surgery.

General physical examination findings were normal. Her body weight was 74 kg, height was 162cm, and all other vital signs were stable. Breast examination revealed Tanner stage 5 for both breasts, which is typical for her age. Her genitalia examination revealed normal labia majora, labia minora, normal pubic hair development, and external urethral meatus. Blood investigation revealed hemoglobin 12.6 gm%, total leukocyte count (TLC) of 14,400/mm^3^, platelets of 60,000/mm^3^, and peripheral blood karyotype 46, XX. Rest of the laboratory parameters are shown in Table 1. Hormonal level of luteinizing hormone (LH), follicle stimulating hormone (FSH), prolactin, progesterone, and testosterone levels were all normal. Magnetic resonance imaging (MRI) of pelvis revealed agenesis of uterus, cervix and proximal two third of vagina along with agenesis of left kidney.

**Table 1:** Laboratory parameters of the patient.

Test	Result	Unit	Reference range
**Hematology**			
**White Blood Cells** (WBC)	14,400	/cm m	4000–11,000
**Hb**	12.6	gm%	12.5–15.0
**PCV**	34.0	%	37.5–45
**Platelets**	60,000	/cumm	1,50,000–4,00,000
**DLC**			
Neutrophils	70	%	45–75
Lymphocytes	20	%	25–45
Monocytes	00	%	2–10
Eosinophils	10	%	1–6
Basophils	00	%	0–1
**Prothrombin time (PT)**	13	sec	10–12
**International normalized ratio (INR)**	0.95		
**Biochemistry**			
Sugar	5.2	mmol/L	3.8–7.8
Urea	3.1	mmol/L	1.6–7.0
Creatinine	63.0	*Mmol/L*	40–110
Sodium	140.0	mEq/L	135–146
Potassium	4.2	mEq/L	3.5–5.2
**Serological Exam**			
Human Immuno deficiency virus antibody (HIV Ab)	Non-Reactive		
Hepatitis B surface antigen (HBsAg)	Non-Reactive		
Hepatitis C virus antibody (HCV Ab)	Non-Reactive		

Following this she was diagnosed with MRKH syndrome with agenesis of left kidney. She was given psychological counseling and was planned for vaginoplasty. Split thickness skin graft was taken from anteromedial aspect of right thigh. Blunt dissection was done and space was created on either side of the vestibule. Intervening tissue were resected and neovagina was created. The mold was wrapped with the skin graft and it was inserted inside the neovagina. Per rectal examination was done in which no abnormality was detected.

## 3 Discussion

Mayer-Rokitansky-Kuster-Hauser syndrome (mullerian agenesis) is a spectrum of congenital anomalies with no known exact cause however mutation in **WNT4** gene is the cause of Mullerian aplasia and hyperandrogenic [4]. There is utero-vaginal agenesis in women with normal ovaries and secondary sexual characteristics [1]. The uterus may be rudimentary as bilateral and non-cannulated muscular buds with normal fallopian tubes and normal ovaries with normal endocrine and cytogenetic evaluations [5]. It has autosomal dominant mode of inheritance. It is caused due to abnormal development of mullerian ducts and the organs like kidney also develop from same embryonic tissue due to which they are involved. MRKH has similarity with Mullerian duct aplasia-renal agenesis-cervicothoracic somite dysplasia (MURCS) association in abnormal sexual development as cervicothoracic somite anomalies, unilateral renal agenesis and conductive deafness are seen in both [6].

Our patient presented with mullerian agenesis and agenesis of left kidney which means MRKH type 2 (MURCS) association. MRKH type 2 may involve the upper urinary tract, the skeleton and conductive system; and cardiac defects are rarely seen [1]. Urinary tract malformations are seen in 40% of the cases which mainly includes unilateral renal agenesis, hypo plastic kidneys, horse shoe shaped kidneys and hydronephrosis [1]. At present we are reporting a case where there is absence of left kidney. There is no visible chromosomal anomaly. Skeletal anomalies are seen in 30–40% of the cases [1]. It includes scoliosis, isolated vertebral anomalies, ribs malformations, and spina bifida. Face and limb extremities involvement is less frequently seen [1]. Limb abnormality includes ectrodactyly, absence of right thumb, duplicated thumb [1]. Auditory defects are seen in 25% of MHRC type 2 cases with conductive deafness due to stapedial ankyloses due to middle ear malformations or sensorineural defects of varying severity [1]. Cardiac malformations are rarely seen but may present with atrial septal defect, conotruncal defects like pulmonary valvular stenosis or tetralogy of fallot if heart is involved [1].

The diagnosis of MRKH mainly depends on imaging study. Transabdominal ultrasonography is the first line investigation but abdomino-pelvic MRI gives more precise and clear information than the prior [7]. So, we suggested our patient to do MRI even though she had done ultrasonography earlier. The differential diagnosis includes congenital vaginal agenesis, low transverse vaginal septum, androgen insensitivity, and imperforate hymen [7].

Young women diagnosed with MRKH suffer from anxiety and mental stress after knowing not having uterus and vagina. So, patient counseling is the first step done before any treatment which was done in our case Treatment involves both non-surgical creation of neo-vagina as well as surgical creation of neo-vagina. In non-surgical methods, Franck's dilator method is done in which vaginal dilators (Hegar candles) are placed on perineal dimple for at least 20 minutes a day to increase the length and diameter of vagina. This process has success rate varying from 78% to 92% and is a first line therapy as it is non-invasive method and often successful. Surgical methods include the Abbe-McIndoe operation, sigmoidal colpoplasty, and the Vecchietti operation [6]. Our patient has undergone Abbe-McIndoe technique of vaginoplasty. This was performed after discussion of various modalities of treatment with the patient and her preference as this technique of vaginoplasty to other modalities of treatment.

In our patient, split thickness skin graft was taken from antero-medial aspect of middle left thigh and a pouch was created between rectum and urethra. Then a mold covered with skin graft was placed into the space and sutured. Then, dilator was inserted into the vagina for 3 months, so that neo-vaginal stenosis doesn't occur and there was long term dilatation of neo-vagina.

## 4 Conclusion

In summary, we here present very rare case of our region of type 2 MRKH syndrome with normal external genitalia but absent uterus, vagina and cervix. This case presents that MRKH syndrome can occur with normal endocrine function and secondary sexual characteristics. Surgical correction by creating a neovagina is a good treatment method in young females for sexual intercourse. Further studies are required to understand the different aspects of MRKHS.

## Provenance and peer review

Not commissioned, externally peer-reviewed.

## Ethical approval

None.

## Please state any sources of funding for your research

No funding was received for the study.

## Author contribution

UR, SA, SS, wrote the original draft reviewed and edited the original manuscript. RD, SP, SS, SKS, RG, AA, and BG reviewed and edited the manuscript and were in charge of the case.

## Please state any conflicts of interest

Authors have no conflict of interest to declare.

## Registration of research studies

1. Name of the registry: None
2. Unique Identifying number or registration ID: None
3. Hyperlink to your specific registration (must be publicly accessible and will be checked): None

## Guarantor

Dr Umesh Ray.

## Consent

Written informed consent was obtained from the patient for publication of this case report and accompanying images. A copy of the written consent is available for review by the Editor-in-Chief of this journal on request.
